# Reliquiæ Houstounianæ: seu Plantarum in America meridionali a Gulielmo Houstoun, M. D. R. S. S. collectarum Icones manu propria ære incisæ; cum descriptionibus e schedis ejusdem in bibliotheca Josephi Banks, Baroneti, R. S. P. asservatis

**Published:** 1784

**Authors:** 


III. *Reliquiæ Houſtounianæ: ſeu Plantarum in America meridionali a* Gulielmo Houſtoun, *M. D. R. S. S. collectarum Icones manu propria ære inciſæ; cum deſcriptionibus e ſchedis ejuſdem in bibliotheca* Joſephi Banks, *Baroneti, R. S. P. aſſervatis.* 4to Londini.

DR. Houſtoun, an ingenious Scotch phyſician, who was employed by the truſtees of the province of Georgia to collect the moſt uſeful plants of the Weſt Indies for that colony, engraved engraved many of the plants he collected, with his own hand, and defcribed them according to Tournefort's method.

After his death, which happened at Jamaica in 1733, his plants, drawings, and engravings came into the poffeffion of the late Mr. Philip Miller. Linnæus, during his ftay in England, faw this collection; and it feems likely that Mr. Clifford received fome of the plants, as feveral of them are defcribed by Linnæus in his *Hortus Cliffortianus.* After Mr. Miller's death, the whole of his collection, including that of Dr. Houftoun, was purchafed by Sir Jofeph Banks, Bart. the learned Prefident of the Royal Society, a gentleman to whom every branch of fcience, but particularly Natural Hiftory, is eminently indebted; and to him the thanks of the botanic reader are due for the prefent work, which has been printed at his expence, and liberally diftributed to learned focieties, public libraries, and botanifts in different parts of Europe.

The work confifts of the whole of Dr. Houftoun's engravings, which are 26 in number. The defcriptions are copied from his MS. and to the name of Houftoun are in general added thofe of Linnæus or Miller. As no co-

 pies pies of the work are fold, we fhall here mention the names of the plants it contains; viz. 1. Jufticia — *Jufticia fcorpioides* Linn. — 2. Kempfera — *Verbena curaffavica* Linn. — 3. Buddleja — *Buddlea Americana* Linn. — 4. Randia — *Randia mitis* Linn. — 5. Ammania — *Ammannia latifolia* Linn. — 6. not named by Houftoun — *Cordia Gerafchanthus* Linn. — 7. Gronovia — *Gronovia fcandens* Linn. — 8. Michelia. — 9. Ricardia — *Richardia fcabra* Linn. — 10. Martynia — *Martynia annua* Linn. — 11. Petrea — *Petrea volubilis* Linn. — 12. Lippia — *Lippia Americana* Linn. — 13. Douglaffia — *Volkameria aculeata* Linn. — 14. Guazuma — *Theobroma Guazuma* Linn. — 15. Juffievia — *Jatropha herbacea* Linn. — 16. Aloe Americana, arboribus innafcens; foliis latis, membranaceis, ad margines fpinofis. — 17. Caffia Americana tetraphylla — *Caffia fruticofa* Mill. Dict. — 18. After Americanus, foliis pinnatis et ferratis, floribus aurantiis — *After aurantius* Linn. — 19. After Saturejæ foliis, conjugatis et pilofis, flore luteo — *Inula faturejaoides* Mill. Dict. — 20. Chryfanthemum Americanum, humile; Origani folio, flore luteo. — 21. Caltha Americana, foliis lanciniatis, flore luteo — *Melampodium Americanum* Linn.

Linn. — 22. Ricinoides folio ſubrotundo, ſerrato; fructu parvo, conglomerato — *Croton glandulosum* Linn. — 23. Mimoſa non ſpinoſa, paluſtris, et herbacea, procumbens; flore luteo pleno — *Mimoſa plena* Linn. — 24. Mimoſa fruticoſa, ſpinoſa; ſiliquis latis, hirſutis et articulatis — *Mimoſa aſperata* Linn. — 25. Mimoſa herbacea, procumbens et ſpinoſa; caule quadrangulo, ſiliquis quadrivalvibus — *Mimoſa quadrivalvis* Linn. — The twenty-ſixth plate, which cloſes the work, exhibits a ſpecies of mimoſa, of which no deſcription is to be found amongſt Dr. Houſtoun's papers.

